# Understanding Patient Experiences: A Mixed-Methods Study on Barriers and Facilitators to TB Care-Seeking in South Africa

**DOI:** 10.3390/tropicalmed10100283

**Published:** 2025-10-03

**Authors:** Farzana Sathar, Claire du Toit, Violet Chihota, Salome Charalambous, Denise Evans, Candice Chetty-Makkan

**Affiliations:** 1 The Aurum Institute, 33 Wrench Road, Johannesburg 1613, South Africa; clairedtt5@gmail.com (C.d.T.); vchihota@auruminstitute.org (V.C.); scharalambous@auruminstitute.org (S.C.); 2 School of Public Health, University of Witwatersrand, Johannesburg 2193, South Africa; 3 Division of Infectious Diseases, Department of Medicine, Vanderbilt University Medical Center, Nashville, TN 37232, USA; 4 Health Economics and Epidemiology Research Office, Faculty of Health Sciences, University of the Witwatersrand, Johannesburg 2193, South Africa; devans@heroza.org (D.E.); cchetty@heroza.org (C.C.-M.)

**Keywords:** health-seeking, barriers, stigma, tuberculosis, mixed-methods, South Africa

## Abstract

Introduction: Tuberculosis (TB) remains a public health concern, and people at risk for TB are hesitant to seek care. The first South African National TB prevalence survey, conducted in 2017–2019, found that most participants with TB symptoms did not seek care for TB. In 2022, an estimated 23% of people with TB in South Africa were undiagnosed, contributing to the country’s burden of “missing” TB cases. This study explores health-seeking behaviour among people with TB (PwTB) in South Africa, focussing on barriers and facilitators to care-seeking and the quantification of TB-related stigma from a patient and community perspective. Methods: We conducted a mixed-method study in the City of Johannesburg (COJ) Metropolitan Municipality from February to March 2022. PwTB aged 18 and older initiating TB treatment for microbiologically confirmed pulmonary TB were recruited from three primary healthcare facilities in the COJ. After providing written informed consent, they participated in a one-time, in-depth, face-to-face interview. The interviews were digitally recorded and conducted by trained facilitators. We used thematic analysis with deductive approaches to develop themes. We used the Van Rie TB stigma assessment scale to quantify perceived stigma. Results: We interviewed 23 PwTB with an overall median age of 39 years and 14 (61%) males. Patient-level barriers to accessing TB care included visiting traditional healers and pharmacists before their TB diagnosis; wrong or missed diagnosis by private doctors; work commitments; scarcity of resources to attend the clinic or walk long distances; perceived and experienced stigma; and a lack of TB knowledge. Facility-level barriers included long clinic queues and uncertainty about where to receive TB care in the clinic. Facilitators for TB care-seeking included being in contact with someone who had TB, receiving encouragement from family, or having knowledge about TB transmission and early diagnosis. The overall median total stigma score among 21 PwTB was 53 (IQR: 46–63), with median community and patient stigma scores of 25 (IQR: 22–30) and 31 (IQR: 21–36), respectively. Conclusions: We found important considerations for the TB programme to improve the uptake of services. Since PwTB consult elsewhere before visiting a facility for TB care, TB programmes could establish private–public partnerships. TB programmes could also increase TB awareness in the community, especially among males, and mobile clinics could be considered to assist with TB case detection and treatment provision. Applying behavioural design techniques and co-designing interventions with patients and providers could improve TB health-seeking behaviours.

## 1. Introduction

In 2022, the World Health Organization (WHO) estimated that approximately 23% of South Africans who had TB were not diagnosed [1]. Most public health systems use passive case finding that relies on a symptomatic individual accessing a health facility to be diagnosed with TB [2]. If the health facility is not accessible or the person does not seek care, this delays the TB diagnosis. A delay in TB diagnosis and treatment may increase individual morbidity and mortality further driving the spread of TB within the community. We recently completed work in South Africa that illustrated the median time from onset of the first TB symptom to starting TB treatment was 5 weeks [3]. This finding prompted an interest in understanding the reasons for delays in seeking care among people with TB (PwTB).

There are multiple patient-level barriers to accessing TB care that have been identified in East and West Africa. These include TB/health literacy levels, food and transport costs, income, stigma, community resistance, and rural residence [4,5,6]. Health-seeking behaviour among PwTB included visits to pharmacists [7,8,9], traditional healers [7,8,9,10,11], herbalists [9], private sector [9,11], and the public sector [9] before a clinic visit. Studies in South Africa found that the delay in diagnosing TB is caused by multiple care-seeking, a provider’s failure to diagnose TB at first contact, and a lack of suspicion of TB [12,13]. There are also multiple facility-level barriers to accessing care that have been previously identified. These include long distances to the clinic, a shortage of healthcare workers, lack of trained healthcare workers, health service delay, and inadequate basic infrastructure [4,5,6,14,15]. However, there is limited literature evaluating the perceived barriers to accessing care and facilitators for TB care-seeking among PwTB in South Africa. South Africa may also be different to these East and West African countries due to its established private sector and higher economic development.

Although access to TB care can be impacted by various factors, TB stigma continues to be a major social factor and is a known barrier to seeking care [16]. A study in Thailand found that PwTB who reported a higher perceived TB stigma demonstrated longer delays in seeking care [17]. Health related stigma has been defined as “a social process or related personal experience characterized by exclusion, rejection, blame, or devaluation that results from experience or reasonable anticipation of an adverse social judgment about a person or group identified with a particular health problem” [18]. Therefore, understanding personal experiences and perceptions of PwTB is critical to inform public health interventions to improve health-seeking behaviour. A previous study in Nigeria found that TB stigma was associated with older age, low socioeconomic status, a level of education below the secondary level, disclosure of TB status, history of weight loss, and previous smoking and alcohol history [19]. However, this study did not use a validated tool to measure stigma.

In 2018, the first South African national TB prevalence survey found that the majority (67%) of survey participants who had TB symptoms did not seek care for their TB symptoms [20]. Some reasons for not seeking care included feeling that their symptoms were not serious enough, insufficient money for transport, and long distances to the healthcare facility. A quantitative assessment of the national TB prevalence survey data found that older people, those residing in rural areas, those having a history of TB, and diabetics were more likely to seek care [21]. Those who were less likely to seek care included those who were HIV-negative, had an unknown HIV status, were cigarette smokers, and alcohol consumers [21]. Since the national TB prevalence survey also recommended that there should be qualitative research to understand the reasons for delayed TB care-seeking, we explored the perceived barriers and facilitators to TB care-seeking among PwTB in South Africa.

## 2. Methods

### 2.1. Study Setting and Design

This study among PwTB occurred between February and March 2022 in three primary healthcare clinics offering TB services in urban Johannesburg, South Africa.

We used a convergent parallel mixed-methods design, collecting both stigma survey data and in-depth interviews during the same period. Each dataset was analyzed on its own first, and we then brought the findings together to see where they converged or added nuance to one another. This approach gave us a fuller picture of the barriers and facilitators to TB care-seeking. The intent was to use the quantitative stigma scores to help frame and enrich the qualitative stories and to improve confidence in our findings through triangulation.

At the start of the study, we worked closely with the nurse responsible for initiating TB treatment to set up a referral system for recruitment. The nurse identified patients aged 18 years and older with microbiologically confirmed pulmonary TB (Xpert MTB/RIF or culture-positive) who were within five days of starting treatment. The study team then confirmed each patient’s eligibility and willingness to participate. We used convenience sampling to recruit participants, aiming for a mix of genders and ages to capture a range of experiences. Eligible PwTB were invited to participate in the study and provided written informed consent for both participation and audio recording. Participant information and consent forms were available in English, isiZulu, and Sesotho. Trained research staff conducted face-to-face interviews at enrolment in a private space at the healthcare facility to reduce social desirability bias and maintain confidentiality.

### 2.2. Data Collection

Quantitative and qualitative data was collected from the same participants.

Quantitative: A structured questionnaire was used to collect participant socio-demographic information. TB-related stigma was measured using the Van Rie TB Stigma Scale [22], a validated instrument designed to assess perceived and experienced stigma among individuals diagnosed with TB. The scale consists of two subscales: Community Perspectives on TB Stigma (11 items) and Patient Perspectives of TB Stigma (12 items). Each item is scored on a 4-point Likert scale ranging from “strongly disagree” to “strongly agree”, with higher scores indicating greater levels of stigma. Interviewers documented responses on paper forms, which were subsequently entered into Excel for analysis.

Qualitative: Interviews were conducted with 23 PwTB using an in-depth interview (IDI) guide (Supplementary Materials). This sample was sufficient to notice a recurrence in themes to reach saturation. Probes on the IDI guide explored TB knowledge, patterns of health-seeking behaviour, and perceived barriers and facilitators to accessing services before diagnosis and during TB treatment, as well as health worker and family support during TB diagnosis and treatment.

We considered reflexivity and positionality carefully during data collection. Interviews were carried out by female research staff with postgraduate training and prior qualitative research experience but who were not part of the clinic teams. At the start of each interview, they introduced themselves as external researchers to help reduce any sense of hierarchy and encourage participants to speak freely. Interviewers took time to build rapport and aimed to stay neutral during conversations to avoid influencing what participants chose to share. Two research staff were trained in qualitative data collection procedures before conducting IDIs to ensure that IDIs were administered in a standardized way. Each participant had one IDI at the clinic with no repeat interviews. Participants were reimbursed with ZAR 150 (less than 10 USD) for their time and effort. On average, the IDIs took approximately 30 min and sessions were audio-recorded. Interviews were conducted in English, isiZulu, and Sesotho. Research staff first transcribed the recordings verbatim in the language in which they were conducted, then translated the isiZulu and Sesotho transcripts into English as needed. A second bilingual team member reviewed the translations for accuracy, and any discrepancies were discussed and resolved to preserve meaning and cultural nuance. English transcripts were used for coding and analysis. The transcripts were quality checked and de-identified before analysis. Data collection and analysis adhered to the COREQ (COnsolidated criteria for REporting Qualitative research) guidelines.

### 2.3. Data Analysis

Quantitative and qualitative datasets were linked using the participant’s unique study ID that was assigned at enrolment.

Quantitative: Data was entered in Microsoft Excel and imported to Stata version 16 (StataCorp, College Station, TX, USA) for analysis. Descriptive statistics were used to summarize participant characteristics, with categorical variables presented as frequencies and percentages and continuous variables summarized using means and standard deviations or medians and interquartile ranges, as appropriate. For descriptive analysis, stigma scores were summarized using medians and interquartile ranges. Comparisons of stigma scores across participant subgroups (e.g., sex, education, etc.) were conducted using *t*-tests or non-parametric tests (e.g., Wilcoxon rank-sum test) as appropriate.

Comparisons across subgroups were conducted using chi-square tests for categorical variables and *t*-tests or Wilcoxon rank-sum tests for continuous variables, depending on data distribution.

Responses to the Van Rie TB Stigma Scale were scored according to the original scoring algorithm. Total stigma scores were calculated for each subscale and as an overall composite (maximum score of 92), with possible scores ranging from 11 to 44 for community stigma and 12 to 48 for patient stigma (a higher score indicates greater stigma). Internal consistency of the subscales was assessed using Cronbach’s alpha to confirm that the tool performed reliably in our study population, as psychometric properties can vary across settings.

Qualitative: Two data coders (FS and CDT), with Master’s level qualifications, coded the English transcripts using NVivo version 14 software. The senior author (CCM) is an experienced qualitative researcher with a doctoral degree and supervised the data analysis. The data coders first did a quality check on the transcripts to ensure the removal of all personal identifiers. The data analysts coded a sample of the transcripts, and the senior author qualitatively checked inter-rater reliability between the coders. Further coding occurred after the codebook was revised during multiple round table discussions maintaining a reflexive approach. We tracked the coding process using a diary of steps and used thematic analysis with deductive approaches to derive the themes.

To strengthen the credibility of our findings, any differences in coding were discussed until the team reached consensus, and themes were refined in consultation with the senior author during peer debriefing. We also kept a written record of analytic decisions, which added transparency to the process.

**Figure 1:**
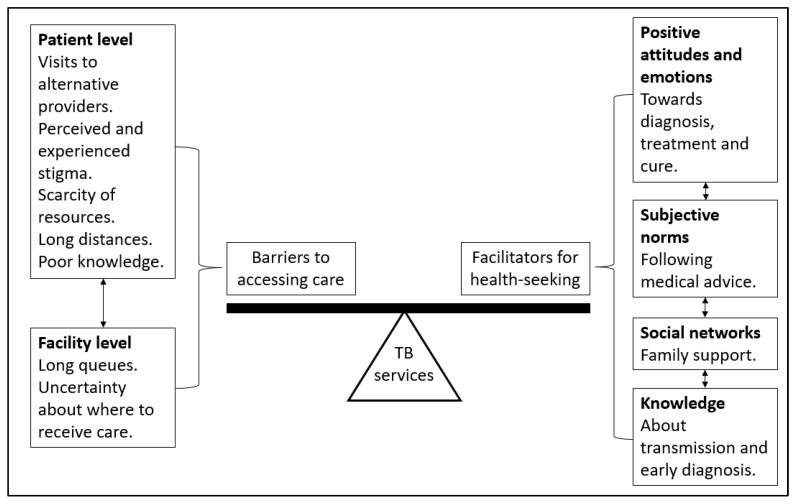
TB care-seeking model as adapted from Nyasulu et al. [23].

In 2018, Nyasulu et al. developed a model of early diagnosis-seeking behaviour [23]. Figure 1 is an adaptation of their framework, which we used for this analysis. Our adaptation included patient- and facility-level barriers to accessing care and person-centred facilitators for health-seeking. The balance between health-seeking and overcoming barriers to accessing care leads to utilizing TB services. For example, patients need to be willing to seek healthcare but also need to visit an appropriate TB service provider.

### 2.4. Ethical Considerations

Ethics approval was obtained from the University of the Witwatersrand Human Research Ethics Committee (HREC). All participants provided written informed consent in their preferred language (English, isiZulu, or Sesotho) after being informed that participation was voluntary and that they could decline or withdraw at any point without affecting their clinical care. Interviews were conducted in private spaces to ensure confidentiality, and all study documents used study IDs rather than personal identifiers. Participants who expressed distress or psychosocial concerns during interviews were referred to the clinic nurse for appropriate support or counselling.

## 3. Results

Quantitative: We included 23 PwTB, of which the median age was 39 years [IQR: 27–54] and 61% were male. Participants were primarily single (48%), unemployed (65%), and 30% had completed secondary/high school. The median distance that the PwTB lived from the clinic was 9 kilometres (IQR: 5–17). The median duration from the onset of the first TB symptom (cough) to starting TB treatment was 2.5 weeks (IQR: 1–4).

Two participants chose not to complete the stigma questionnaire following their interviews, which resulted in missing scores, but this did not affect their inclusion in the qualitative analysis. The overall median total stigma score among 21 PwTB was 53 (IQR: 46–63), with median community and patient stigma scores of 25 (IQR: 22–30) and 31 (IQR: 21–36), respectively (Table 1). For each demographic variable, we assessed the difference between the medians of the respective categories and found that there were no statistically significant differences. Cronbach’s alpha for the TB Stigma Scale was 0.86, indicating a very good level of internal consistency.

**Figure 2:**
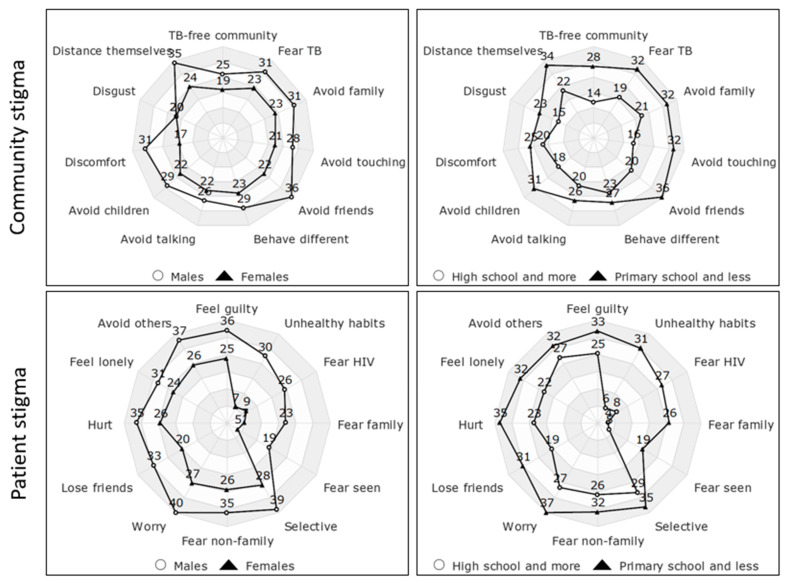
Community and patient TB stigma scores by demographics.

When looking at the responses to the individual questions on the community and patient perspective questions (Figure 2), males scored higher than females, and those with primary or no schooling scored higher than those with high school education.

Qualitative: We describe two themes from the analysis: (i) barriers to accessing TB care and (ii) facilitators for health-seeking, pre- and post being diagnosed with TB (Table 2).

### 3.1. Theme 1: Barriers to Accessing TB Care

#### 3.1.1. Pre-TB Diagnosis

PwTB sought care at different providers before being diagnosed with TB. These consultations included but were not limited to seeking care from a traditional healer, private doctor, or pharmacist. PwTB reported that they treated their symptoms with home remedies, traditional medicine, or over-the-counter medications. Reasons for first visiting other providers include a lack of money and lack of suspicion of a TB diagnosis.

“*I did not start at the clinic because you know as black people we sometimes believe in witches*.”

(Male, 40 years, stigma score = 63)

“*Then I went to see a guy who is a traditional healer, I went to see him and he gave me something for steaming and bathing*.”

(Male, 38 years, stigma score = 77)

“*After coming from the doctor, I found out that I didn’t have the money to buy the things that he had prescribed*.”

(Male, 71 years, stigma score = 53)

“*The first place I went to was {} the chemist, the chemist at the corner next to the garage. I went to the chemist, {} I do not like going to the clinic {} because I do not have money to go to the doctor, {so} I went to the chemist to tell them that it’s just flu*.”

(Male, 38 years, stigma score = 81)

“*Ah I thought it was flu, something minor that will go away, I was busy taking grandpa, saying that it will go away*.”

(Female, 47 years, stigma score = 74)

PwTB mentioned the hassle of walking long distances to the clinic due to lack of transport. A scarcity of resources contributed to them delaying accessing care.

“*Sometimes {I} ask my brother to accompany me, we walk and take shortcuts*.”

(Female, 47 years, stigma score = 74)

“*The distance is too long. Because your feet become painful when you’ve got TB, they have a stabbing sensation at the bottom*.”

(Male, 51 years, stigma score = 60)

“*We struggle with transport this side*.”

(Female, 23 years, stigma score = 61)

Perceived and experienced stigma were identified as barriers to care. Some participants mentioned that they preferred travelling a greater distance to a clinic where they were not known. However, this choice results in increased costs and longer travel times, which in turn create additional barriers to accessing care.

“*Is not too far but I wouldn’t prefer to go to my nearest clinic because there’s people that know me, so when they know that I am going there for this and that, they might look at me in another way, so I prefer going to the clinic that’s far from my community*.”

(Male, 23 years, stigma score = 37)

“*They should not be afraid of me or avoid me because I have TB, like some not wanting to sit with me like we used to before, finding myself alone*.”

(Male, 37 years, stigma score = 46)

“*I know people will say a lot about TB because mostly people they always say because you have TB it means you are HIV positive*.”

(Male, 23 years, stigma score = not available)

“*My only experience was with the neighbour, who was like, oh he’ s got TB, he will pass it to us and I have {seen}{} other people, others even end up staying alone, no one wants to be close to them, when they walk on the street, they move and change directions*.”

(Male, 23 years, stigma score = 37)

#### 3.1.2. Post-TB Diagnosis

Some PwTB did not understand how TB transmission occurs and some had misconceptions about TB transmission. There is a link between knowledge of TB and the correct knowledge about TB transmission, but this does not appear to be associated with seeking healthcare for TB diagnosis and treatment in urban districts of South Africa.

“*For me, the people I stay with, how can I protect them, that is what I want to understand*.”

(Female, 39 years, stigma score = 46)

“*You can get it, when you eat from where they were eating, you can also get it*.”

(Male, 51 years, stigma score = 60)

PwTB were uncertain about where to go when they first visited the primary health facility and expressed frustration with waiting in long queues. PwTB also requested an alternative place to receive their TB medication to avoid long clinic queues. PwTB expressed dissatisfaction with healthcare worker attitudes but also mentioned the need for healthcare workers to provide food to support treatment adherence, offer counselling, and encourage exercise.

“*[Sighs], here the clinic, personally, to the tell you the truth I do not like coming to the clinic because you come, like my first time when I came. You do not know where you have to stand and it is packed {}, you see people who come every day, I think {they} are the ones that know where to go and sometimes the staff {are}{} not friendly. Like they will push you around. Asking you why you are seated here, things like that you see*.”

(Male, 38 years, stigma score = 81)

“*If they can fast track the line {because}{} we are staying too long when we are here to collect or fetch our medication*.”

(Male, 32 years, stigma score = not available)

“*It is getting food before you can get those pills because some of us are no longer working*.”

(Male, 51 years, stigma score = 60)

### 3.2. Theme 2: Facilitators for Health-Seeking

#### 3.2.1. Pre-TB Diagnosis

After noticing that alternative treatments were not improving their symptoms and not having enough money to go to the doctor or pharmacy, PwTB decided to seek care at their nearest public health clinic.

“*But when you start paying attention you then realize that it has been two {}{months} since I have been taking the medicine but nothing has changed*.”

(Male, 40 years, stigma score = 63)

Some PwTB indicated that they were in contact with someone who had TB and started experiencing TB symptoms. This suspicion of TB motivated them to go to the clinic.

“*Yes, because I also got it like that, I got it from the person I was staying with and after some time it got into me*.”

(Male, 40 years, stigma score = 63)

“*Me being in contact with a person who has TB and then like I saw all the symptoms in me as well, {}{yes} that’s why I decided to come to the clinic and get tested and see if I have, so, they have actually just told me that yes indeed I have TB*.”

(Male, 23 years, stigma score = not available)

“*You know my reason for coming here was that I was bringing my grandchild here he was coughing and when I got here. I travelled to the clinic for a week and on the second week I was sent for X-ray, I was later contacted to be told that the results are out and they indicate that I have TB*.”

(Male, 70 years, stigma score = 74)

#### 3.2.2. Post-TB Diagnosis

Family support was a dominant driver for health-seeking behaviour, and the importance of family in helping people remain on treatment was also apparent. PwTB mentioned that their family reminded them to take their TB medication, took care of their basic needs, and provided emotional support.

“*What I can say is that they were reminding me not to skip taking my pills, they always prepared food for me, they did not let me stay alone, they were always with me*.”

(Male, 40 years, stigma score = 63)

“*I also started coughing a lot like my uncle and he suggested that maybe I should also go to the clinic to get tested and then I went*.”

(Male, 38 years, stigma score = 81)

“*So, he makes sure that there is food so that I can take pills, you see things like that*.”

(Female, 36 years, stigma score = 45)

“*I did not have enough money to come here and I did not ask my son in-law, normally when I tell him, he brings me here*.”

(Female, 58 years, stigma score = 61)

“*My mom was always there, whenever I need to talk she is there for me*.”

(Female, 23 years, stigma score = 61)

“*I just need medication and to be reminded to eat my food, that is what I need*.”

(Male, 67 years, stigma score = 53)

Interestingly, following clinical TB diagnosis, PwTB believed that TB treatment would cure them and protect others from contracting TB. PwTB expressed a strong sentiment towards early diagnosis and treatment initiation. PwTB emphasized their satisfaction with the services at the clinic.

“*If you get it [TB] on time, if you find out early that you have TB, I mean its treatable, so, I just want people to you know, educate themselves about TB and once you see the symptoms you should rush to the clinic you know and get help before it’s too late*.”

(Male, 23 years, stigma score = not available)

“*I just want to tell people that whenever you see anything or experience coughing and chest pains and everything, please rush to the clinic, if you go there, trust me they won’t even chase you, they won’t even shout at you, you will actually get the help that you need*.”

(Male, 23 years, stigma score = not available)

“*Like the way I see it is that they are trying to stop it from infecting more people, so you have to start an early treatment so that you don’t affect a large number of people outside*.”

(Male, 25 years, stigma score = 48)

“*TB is curable and if, if you take your medicine or your treatment right*.”

(Male, 32 years, stigma score = not available)

“*So far it is good, I get the treatment, I get the support and there is follow up*.”

(Male, 48 years, stigma score = 46)

Most PwTB experienced mixed emotions towards TB diagnosis and verbalized experiencing the fear of dying from TB.

“*I was so scared first, but I am glad that {} I know what is going on with my {} life now*.”

(Female, 55 years, stigma score = 44)

“*You become scared {}, that it means that you can die, so I was feeling {} what if I die?*”

(Male, 38 years, stigma score = 81)

“*I was thankful that at least I know and I will get help, I was thankful for that*.”

(Female, 47 years, stigma score = 74)

“*Because they said that TB can be cured, {} it didn’t affect me bad*.”

(Male, 40 years, stigma score = 63)

Quantitative stigma scores were interpreted alongside qualitative themes to deepen our understanding of barriers and facilitators to TB care. For Theme 1 (barriers to accessing TB care), participants with higher stigma scores more often described avoiding their nearest clinic, fearing community gossip, or travelling to distant facilities for privacy—illustrating how stigma contributed to delays. In contrast, those with lower stigma scores tended to emphasize structural barriers such as long queues or transport costs rather than the fear of disclosure. For Theme 2 (facilitators for health-seeking), participants with strong family support generally had lower stigma scores and described the encouragement from relatives as an important motivator for starting treatment. Although gender differences in stigma scores were not statistically significant, men frequently spoke about work-related constraints and a fear of judgement by peers, suggesting that qualitative narratives captured nuances not reflected in the quantitative data. Taken together, these findings indicate that stigma interacts with social support, health knowledge, and structural factors to shape care-seeking behaviour.

Although subgroup differences were not statistically significant (Table 1), we observed that males and participants with lower education tended to have higher median stigma scores, and those who first visited traditional healers or pharmacies reported longer delays. These quantitative patterns align with the qualitative narratives in which participants described stigma-related avoidance of local clinics, misconceptions about TB transmission, and reliance on alternative providers as barriers to timely diagnosis.

## 4. Discussion

We gained insights into the perceived barriers (patient- and facility-level) to accessing care which may contribute to delayed TB care-seeking among people living in a resource-limited setting in South Africa. We found that PwTB preferred to first visit traditional healers, private doctors, and pharmacists before going to a facility to access TB services and receive a TB diagnosis. The implications of delaying a TB diagnosis could lead to more advanced disease and transmission. PwTB also end up incurring extra costs on transport and medication when visiting multiple providers. Other self-inflicted extra costs occur when PwTB choose to visit clinics further away from their community to avoid perceived stigma. Although PwTB were initially hesitant to go to the clinic and had misconceptions about the care that they would receive, they were pleasantly surprised by the good service that they received from the facility.

When considering the perspectives of PwTB, they would prefer to avoid the clinic and receive treatment elsewhere. Visits to traditional healers are related to belief systems. A previous study conducted in South Africa found that a rural community believed that TB is a result of breaking cultural rules and that it can only be treated by a traditional healer [24]. Belief systems are difficult to overcome, but healthcare workers and traditional healers should seek constructive solutions together. If clinic queues are too long, then sometimes PwTB would have to return on another day. Another clinic visit required more transport money and time off from work. People in the community could also be hearing about the long clinic queues, which may cause them to avoid seeking care at the clinic, resulting in them visiting other providers. These issues could be addressed with the implementation of decentralized TB services which brings TB care closer to homes. The focus would then be on promoting these decentralized services to encourage TB care-seeking. TB programmes should consider mobile clinics to assist with TB case detection and treatment provision.

Knowledge about TB can reduce stigma and influence appropriate health-seeking behaviour. If we consider the lessons learned from coronavirus disease 2019 (COVID-19), many countries made resources available in response to the COVID-19 outbreak [25]. Technological solutions and mass media campaigns were employed to educate the public. To mitigate misconceptions about TB and improve TB knowledge, digital messaging and social media can be used to communicate evidence-based behaviour change information [25,26]. A quasi-experimental feasibility study conducted in Northern Ireland used a social media platform (Twitter, now known as X) to disseminate information related to skin care in the sun and cancer prevention and found that social media was a feasible platform to deliver public health information and improve knowledge [27]. Collaborative action and co-designing interventions with patients allow for insight into patients’ preferences, which could encourage them to seek care.

The current TB programme focuses mainly on passive case finding. We recommend that TB programmes create private–public partnerships to support TB education and screening among pharmacists, traditional healers, and private doctors. TB programmes could also scale up TB awareness in the community through collaboration with the Department of Education. The private sector should also provide access to free testing and treatment for TB. Individual needs assessments conducted by healthcare workers would deliver a patient-centred holistic package of care.

Understanding different contexts is important to understand access to healthcare. A previous study evaluated the relationship between socioeconomic inequalities and access to healthcare and found that the socioeconomically disadvantaged delay seeking healthcare and are less satisfied with healthcare services [28]. The implementation of the South African National Health Insurance (NHI) aims to achieve universal healthcare access regardless of socioeconomic status. The visible improvements of the public sector could change mental models about the healthcare system, and bridging the private–public divide could eliminate the negative connotations associated with the public health services.

Co-designing and piloting interventions with patients and healthcare providers could improve TB health-seeking behaviours. Inputs from a patient perspective or contextual inquiry can help understand behavioural barriers which motivate patients to visit alternative providers first. Thus, reducing stigma and improving the uptake of TB services. A recent study in Indonesia co-designed a community-based intervention with peers (TB survivors or PwTB) and suggested the following interventions to reduce stigma and depression among PwTB: individual counselling, peer-led group counselling, peer-led support, and community-based TB talks [29]. Similarly, a study conducted in South Africa found that patients requested TB survivors as peer counsellors and community-based education [16].

### Strengths and Limitations

We included a diverse group of PwTB based on clinics, age, and gender that provided different views and perspectives on health-seeking behaviour and barriers to accessing TB care. We interviewed people who were being treated for TB so we could ask about their journey to TB treatment. There are several limitations of this research. Firstly, we did not reach out to patients with TB who did not visit the facility for treatment, those who had not overcome their barriers to receiving care, and we only sampled participants from urban areas in one province. Our findings therefore may not be generalizable to peri-urban or rural settings or provinces with diverse models of service delivery and patient behaviour. Second, convenience sampling was utilized, and while this can lead to selection bias, the small sample size reduced the statistical power for quantitative comparisons. Nonetheless, thematic saturation had been reached in qualitative analysis, affirming credibility of the themes developed. Third, we used education and employment status as proxies for socioeconomic status and did not ask for detailed information about household income or assets, so our findings were not capable of fully investigating the influence of poverty on care-seeking. Fourth, recall bias may have affected participants’ answers regarding the onset of symptoms and care-seeking, and social desirability bias may have affected some of the interview responses. We attempted to counter this by probing and data triangulation but cannot eliminate these entirely. Finally, we sampled subjects with microbiologically documented pulmonary TB, but not subjects with extrapulmonary or clinically diagnosed TB, which could diminish the external validity of our findings to other TB groups. Overall, the findings must be regarded as context-dependent and larger, more representative studies are needed.

## 5. Conclusions

Our study revealed some important considerations for the TB programme to improve the uptake of services. Since PwTB consult elsewhere before visiting a facility for TB care, TB programmes could support education on TB screening and improve partnerships with pharmacists, traditional healers, and private doctors. TB programmes could also scale up TB awareness in the community, especially among males, and consider mobile clinics to assist with TB case detection and treatment provision. Applying behavioural design techniques and co-designing interventions with patients and providers could reduce stigma and improve TB health-seeking behaviours. Behavioural interventions should be designed with a patient-centred approach. By intervening at the provider-level, community-level, and patient-level simultaneously, we could improve health-seeking behaviour among PwTB and facilitate TB care-seeking.

## Figures and Tables

**Table 1:** TB stigma scores among people living with TB.

Demographics		N (%)	Community Stigma Score	*p* Value	Patient Stigma Score	*p* Value	Total Stigma Score	*p* Value
			Median (IQR)		Median (IQR)		Median (IQR)	
Overall score		21	25 (22–30)		31 (21–36)		53 (46–63)	
Sex	Male Female	14 (61) 9 (39)	27 (22–32) 24 (23–27)	0.943	34 (24–37) 23 (19–34)	0.126	57 (47–71) 46 (44–61)	0.188
Marital status	Cohabitation Married Single Widowed	4 (17) 4 (17) 11 (48) 1 (4)	34 (26–37) 22 (21–30) 25 (22–30) 38 (38–38)	0.136	34 (17–43) 31 (25–33) 34 (21–37) 36 (36–36)	0.879	60 (54–77) 53 (46–63) 53 (45–67) 74 (74–74)	0.495
Education	No formal schooling Primary school High school Vocational training	5 (22) 6 (26) 7 (30) 4 (17)	34 (30–34) 29 (22–35) 23 (20–26) 24 (23–25)	0.160	33 (17–43) 36 (34–37) 23 (21–25) 28 (19–36)	0.164	63 (54–77) 64 (53–74) 46 (44–48) 52 (42–61)	0.063
Employment	Yes No	8 (35) 15 (65)	27 (22–30) 25 (22–34)	0.876	28 (18–37) 31 (21–36)	0.755	55 (39–67) 53 (46–61)	0.815
First provider visited	Pharmacy Public clinic Public hospital Traditional healer	3 (15) 12 (60) 2 (10) 3 (15)	25 (19–27) 25 (22–34) 27 (17–37) 30 (30–34)	0.531	34 (18–36) 28 (21–36) 27 (17–36) 37 (33–43)	0.499	61 (37–61) 51 (44–74) 54 (53–54) 67 (63–77)	0.331

**Table 2:** Pre- and post-TB diagnosis barriers and facilitators to TB care-seeking.

Pre-TB Diagnosis	Post-TB Diagnosis
Barriers	
Lack of resources	Long waiting times
Lack of TB suspicion	Staff attitudes
Long distances to the clinic	Stigma
Stigma	
Facilitators	
Symptoms not improving	Being cured with treatment
Lack of resources	Staff attitudes
Suspicion of TB	Family support
Family support	Fear of dying
Fear of dying	

## Data Availability

The data used and/or analyzed during the current study are available from the corresponding author on reasonable request.

## Supplementary Materials

The following supporting information can be downloaded at: https://www.mdpi.com/article/10.3390/tropicalmed10100283/s1.
