# Supplementary material for: Understanding Patient Experiences: A Mixed-Methods Study on Barriers and Facilitators to TB Care-Seeking in South Africa
Source: Trop Med Infect Dis. 2025 Oct 3;10(10):283. doi: 10.3390/tropicalmed10100283 (PMC12567892; doi:10.3390/tropicalmed10100283)
Supplement: Supplementary file 1 [file tropicalmed-10-00283-s001.zip › tropicalmed-3870217-supplementary.pdf]

APPENDIX 1:

IN-DEPTH INTERVIEW GUIDE

A mixed methods study exploring health-seeking behavior among TB patients in South Africa

IN-DEPTH INTERVIEW

Introduction and Ground Rules

1. Obtain written informed consent first, before any data are collected.
2. Interviewer to introduce themselves. Thank you for taking the time to meet with us today. Our names are [*insert names*] ______________ and we would like to talk to you about health seeking for TB. We are doing this project to understand what patients do and where do they go when they experience TB symptoms. We want you to be as open and honest when answering. There are no right or wrong answers in this discussion. Please feel free to tell us what you think.
3. Interviewer to explain the ground rules and terms of confidentiality for the interview:
   - The participant does not have to answer any question they do not want to.
   - The information you share will be handled in confidence.
   - When we report back on the information collected in this discussion, your comments will not be able to be linked to you specifically.
   - We ask that you also agree not to share anything discussed in this room with others.
4. The discussion could take around one hour.
5. Interviewer to inform the interviewee that the in-depth interview will be audio-recorded to make sure that all themes are captured. Turn the audio-recorder on and ask for verbal permission again to audio-record, while the audio-recorder is running to verbally capture consent (this is a double check against the written consent). We will be recording the session because we don't want to miss any of your comments. Although one of us may take some notes while we talk, we can't write fast enough to get everything down on paper. As we are recording, please try to speak loudly so that we don't miss your comments.

APPENDIX 1:

IN-DEPTH INTERVIEW GUIDE

A mixed methods study exploring health-seeking behavior among TB patients in South Africa

Themes to be explored

a) TB history
b) TB knowledge
c) Patterns of health seeking behavior
d) Perceived barriers and facilitators to accessing services
e) Health worker and family support during TB diagnosis
f) Perceived barriers and facilitators to accessing support during TB diagnosis
g) Preferences for TB patient support

Time started (HHMM):

Questions

TB history

1. *Have you had TB before this?*
    - *If yes, how long ago was it?*
    - *If yes, how many times?*
2. *Have you had contact with someone who has had a positive TB diagnosis in the past 6 months? in the past 12 months?*

TB knowledge

3. Please describe to me your thoughts on TB?
    - *Do you believe that TB exists?*
    - *Do you believe that you have TB?*
    - *How can you become infected with TB?*
    - *What are the symptoms of TB?*
    - *Do you believe that TB can be treated?*
    - *If you do not get treatment, will you infect others?*

Patterns of health seeking behavior

APPENDIX 1:

IN-DEPTH INTERVIEW GUIDE

A mixed methods study exploring health-seeking behavior among TB patients in South Africa

4. Tell me your story from when you first experienced signs or symptoms of TB? What did you do? Where did you go?

    - *How did you feel during this time?*
    - *How did you feel when you were diagnosed with TB?*
    - *(If not public health clinic), why did you not come to your public health clinic when your symptoms first started?*
    - *What made you come to the public health clinic for diagnosis and treatment?*
    - *Can you tell me about your experience accessing TB services here at the public health clinic?*

Perceived barriers and facilitators to accessing services

5. What would stop you from seeking health care for TB at your nearest health clinic?

    - *Probe: Health service barriers (distance to clinic, unfriendly staff at the facility etc.)*
    - *Probe: Personal barriers - which include logistical issues to getting to the clinic (e.g. time, cost, distance to clinic, job) and other barriers like stigma, severity of symptoms*
    - *Are you afraid of what people might say if they find out that you have TB?*
    - *Are you afraid of what others might think if they see you at the clinic?*
    - *Have you experienced stigma from your family, clinic or community which would stop you from seeking care?*

6. What would you need in order to visit the clinic / health care facility?

APPENDIX 1:

IN-DEPTH INTERVIEW GUIDE

A mixed methods study exploring health-seeking behavior among TB patients in South Africa

Health worker and family support during TB diagnosis

7. Can you tell me about the support you received while you were ill?

Perceived barriers and facilitators to accessing support during TB diagnosis

8. What would stop you from getting this support during your TB diagnosis?

9. What would you need in order to get support during this process?

Preferences for TB patient support

10. What type of support would you like to receive from?
    - Family
    - Health care facility/Clinic
    - Community
11. Please rank the above in order of your preference?

Any other comments

Are there any final thoughts you have about delivery of TB services?

End of session

Now we have come to the end of our discussion. Thank you for your participation. If you have any questions about your study participation, please contact us. Thank you.

Time ended (HHMM):
